# Numerical and functional response of phagotrophic aquatic protists: the ideal experiment—and why we cannot get it

**DOI:** 10.3389/fmicb.2025.1559802

**Published:** 2025-06-10

**Authors:** Thomas Weisse

**Affiliations:** Research Department for Limnology, University of Innsbruck, Mondsee, Austria

**Keywords:** functional ecology, experimental design, noise and bias, growth rates, ingestion rates, ciliates

## Abstract

Protists are paramount for biogeochemical cycling in every aquatic ecosystem due to their vast population sizes and physiological versatility. Numerical response (NR) and functional response (FR) experiments are cornerstones of trait-based functional ecology and are increasingly studied experimentally with phagotrophic aquatic protists. Such experiments provide estimates of protist growth, production and consumption rates in relation to biotic (food supply) and abiotic variables (e.g., temperature, pH, and salinity) that can be used in mathematical models of ecosystem dynamics. Until now, NR and FR experiments lack standardization and are subject to potential pitfalls that received little attention in the literature. It is a common misconception that an experimental investigation of a phagotrophic protist’s growth and ingestion rates represents a single experiment with replication. I demonstrate that a typical NR or FR experiment consists of a series of individual experiments in which not only the experimental target variable (food, i.e., prey abundance or biomass) changes but also other factors (physiological conditions of prey and predator, nutrient levels, unwanted contaminants) vary that may affect the experimental outcome. Standardizing all variables affecting a series of NR and FR experiments is virtually impossible. I further explain why FR experiments are more prone to experimental bias than NR experiments. Since it is principally impossible to perform an “ideal” NR or FR experiment, fulfilling all criteria of experimental standardization, the goal is to reduce the “noise” to obtain statistically significant and reproducible results. To this end, I provide guidelines that may help achieve this goal in future studies.

## 1 Introduction

### 1.1 Functional ecology of aquatic protists

Protist ecology has increasingly shifted from a taxonomic-oriented approach towards an ataxonomic, trait-based functional approach over the past decades (Fournier et al., 2012; Litchman and Klausmeier, 2008; McGill et al., 2006; Weisse et al., 2016a). This is because key processes in aquatic and terrestrial ecosystems usually depend on the functional performance of groups of similar organisms. Individual species may be replaced by others dwelling in the same habitat without any apparent effect at the ecosystem level. For instance, resource supply strongly affects primary production, while the presence or absence of a given species is generally of little overall importance. Exceptions to this rule are some keystone species (Paine, 1969; Paine, 1995) whose presence can alter whole ecosystem dynamics. Keystone species are primarily known from macroorganisms. Among protists, most species do not seem to be functionally unique, i.e., if one species is lost, its role can usually be filled by other species. This functional redundancy provides buffering capacity at the ecosystem level, enabling stable ecosystem functions (Caron and Countway, 2009; Fuhrman, 2009). Until now, the vast molecular diversity revealed by PCR-based approaches has not been reflected by an apparent increase in ecological functions. However, single-cell genome sequencing, metagenomics and metatranscriptomics are increasingly used to detect new metabolic pathways and improve the understanding of the protists’ functional role in the ecosystem context (Weisse and Montagnes, 2022 and references therein).

Functional ecology seeks to identify and parameterize key processes such as consumption, production, and remineralization rates in the ecosystem context. These processes are studied under *in situ* conditions in the field or, for pragmatic reasons, more often under simulated *in situ* conditions in the laboratory. In either case, it is impossible to investigate each species’ performance and interactions in full detail. Therefore, like their colleagues studying macroorganisms, experimentally working protistologists increasingly focus on investigating model organisms (Montagnes et al., 2012; Weisse, 2006) that represent major functional traits that protists have in the ecosystem context. Ideally, an experiment with a suitable model organism should yield major functional traits with realistic parameter estimates. Numerical response (NR) and functional response (FR) experiments fulfill these criteria (Weisse, 2017), providing estimates of growth, production and feeding rates in relation to biotic (food supply) and abiotic variables (e.g., temperature, pH, and salinity) that can be used in mathematical models on ecosystem dynamics. Such food web models *sensu lato* are instrumental in the context of climate change, predicting future scenarios under altered thermal regimes in terrestrial and freshwater environments (Elliott, 2012; Montagnes et al., 2008) or increasing acidification in the ocean (Poloczanska et al., 2013; Riebesell and Tortell, 2011).

Since NR and FR experiments represent a cornerstone of functional (protist) ecology, this article critically evaluates the pros and cons of these approaches and provides guidelines for future experimental work. The focus is on the experimental design because several competent reviews have been published on the rationale and curve fitting of numerical and functional responses, including derivations of the inherent equations and interpretation of the parameters’ biological meaning (DeLong, 2021; Li and Montagnes, 2015; Montagnes and Berges, 2004; Okuyama, 2013; Okuyama and Ruyle, 2011). Although the present article primarily reviews previous work, I present an as-yet unpublished problematic case study with a mixotrophic freshwater ciliate to illustrate the main issues and provide a template for future research. I will demonstrate that it is not only stochasticity (“noise”) but bias (i.e., systematic error) that may affect the experimental outcome. However, despite these caveats, the gain from the experimental results far outweighs the inherent pitfalls.

This integrative work, focusing on the practical approach in a well-defined theoretical framework, does not only address protistologists. This is because working experimentally with protists also appeals to researchers who do not focus on unicellular organisms. An increasing party of ecologists and evolutionary biologists take advantage of the fact that many protists are easy to cultivate and manipulate, reach higher cell numbers, and have shorter generation times than macroorganisms (Weisse and Montagnes, 2022). Although protists have been used more often in recent years to address broader biological, macroecological and macroevolutionary issues (Chaine et al., 2010; Jacob et al., 2017; Laurent et al., 2020; Montagnes et al., 2012), this approach has a long tradition. For instance, Gause (1934) used *Paramecium* in his classical competitive exclusion experiments. Since many constraints inherent in FR and NR experiments similarly apply to other experiments with and without protists, I expect that the general considerations and guidelines I provide may also be helpful for experimentally working “non-protistologists.”

### 1.2 The framework: numerical and functional responses

The general design of NR and FR experiments with aquatic protists has been reviewed several times (Li et al., 2013; Montagnes, 2013; Weisse et al., 2016a) and shall not be repeated here in detail. Briefly, NR investigates the specific growth rate of a heterotrophic or mixotrophic protist population depending on its food supply; similarly, FR experiments relate the feeding rates of a protist predator to its prey abundance or biomass (see Table 1 for graphical illustration and calculation of experimental results). Both experimental types yield four immediate parameters (i.e., variables; Table 1), each of which can be used in mathematical models.

**TABLE 1:** Characteristics of the numerical response (NR) and functional response (FR).

Parameter	Equation (no.)	Variables	NR and FR curve and comments
**A. Numerical response**			
Specific growth rate (*r*) of the predator	$r=\ln\,\left(\frac{N_{t}}{N_{0}}\right)\,\frac{1}{t}$ (1)	Initial (*N*_0_) and final (*N*_*t*_) predator cell numbers (mL^–1^ or L^–1^); *t* = experimental duration (d)	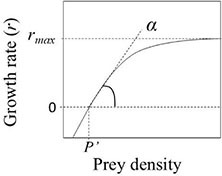 Figure A. The numerical response curve. Prey density can be expressed as abundance or biomass.
Geometric mean prey concentration (*P*)	$P=\frac{\left(P_{t}-P_{0}\right)}{\ln\,\left(P_{t}-P_{0}\right)}$ (2)	Initial (*P*_0_) and final (*P*_*t*_) prey densities (mL^–1^ or L^–1^) in experimental containers	
Numerical response	$r=\frac{r_{\max}\,\left(P-P^{{}^{\prime }}\right)}{k_{2}\,\left(P-P^{{}^{\prime }}\right)}$ (3)	*r*_max_ = maximum growth rate (d^–1^); *P*′ = the *x*-axis intercept (i.e., the threshold prey density, where *r* = 0); *k*_2_ is a constant (mg C L^–1^); in Fig. A (right), the searching rate α corresponds to the initial slope of the curve	
**B. Functional response**			
Ingestion rate of the predator (*I*, ng C Cil^–1^ d^–^)	$I=\frac{P\,g\,m}{R_{m}}$ (7)	*g* = grazing rate (d^–1^, see below); *m* = cellular prey biomass (ng C cell^–1^); *Rm* = geometric mean predator abundance (mL^–1^)	If not multiplied by *m*, *I* can be expressed as prey cells eaten predator^–1^ d^–1^
Grazing rate (d^–1^)	$g=\left(\ln\,\left(\frac{C_{t}}{C_{0}}\right)-\ln\,\left(\frac{P_{t}}{P_{0}}\right)\right)\,\frac{1}{t}$ (8)	initial (*C*_0_) and final (*C*_*t*_) prey cell numbers (mL^–1^ or L^–1^) in controls without predators	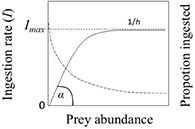 Figure B. Type II functional response. Ingestion rate (solid line) and prey proportion ingested (dashed) vs. prey abundance (or biomass).
Type II functional response	$I=\frac{I_{\max}\,P}{\left(k+P\right)}$ (9)	*I_*max*_* = maximum ingestion rate of the predator (prey cells predator^–1^ d^–1^ or, in terms of biomass, ng C cell^–1^ d^–1^); *k* = half saturation constant (mg C L^–1^)	
**Derived parameters**			
Clearance rate (mL predator^–1^ d^–1^)	$C=\frac{I}{P}$ (10)	*I* and *P* as above	*C* is identical to the proportion ingested (Fig. B)
Gross growth efficiency (GGE)	$\mathrm{GGE}=\frac{r\,M}{I}$ (11)	*M* = cellular biomass of the predator (ng C cell^–1^)	the fraction of prey biomass converted into predator biomass; may also be expressed in terms of cell volume

The numerical response is calculated using the Equations 1−3 (Table 1). While the NR usually follows a rectangular hyperbolic function (Figure A in Table 1), the curve of the FR can take three different types (Holling, 1959). The general form of the FR equation is


(4) $$I=\frac{a\,P^{\theta }}{1+a\,h\,P^{\theta }}$$

where *I* is ingestion rate (prey per predator per time, also known as feeding rate, foraging rate or consumption rate), *P* is prey abundance (or density), θ (Theta) is the shape parameter (a.k.a. the Hill exponent), *h* is the handling time (i.e., the time taken to process a single prey item), and α is the searching rate (*a* is also known as instantaneous rate of discovery, attack rate, capture rate, maximum clearance rate and affinity between the predator and prey; Kalinkat et al., 2023; Montagnes, 2013; Rosenbaum and Rall, 2018). However, with dimensions of area or volume per predator and time, the parameter *a* is not a rate of attack. For this reason, DeLong (2021) suggested renaming α space clearance rate, which is more appropriate. The inverse of *h* is the maximum ingestion rate, *I*_*max*_. Note that Eq. 4 describes the per capita rate of feeding, not the total prey consumption by a predator population in a unit of time.

In the rectilinear type I response, the predator does not spend time handling the prey. Therefore, if θ = 1 and h = 0, *I* increases linearly with prey density (i.e., Eq. 4 is reduced to *I = a P*) at low and moderate prey levels. When a satiating food level is reached (at which *I* = *I*_*max*_), *I* remains constant with further increasing prey density. The type I FR is typical of filter-feeding metazoans but is less common in free-swimming protists (Jeschke et al., 2004).

For Holling’s type II and type III responses, the shape parameter in Eq. 4 determines the non-linearity of the FR curve. If θ = 1, the function is a saturating type II response:


(5) $$I=\frac{a\,P}{1+a\,h\,P}$$

The rectangular hyperbolic FR (Holling’s type II; Figure B in Table 1) is the most widely used model. θ > 1 yields sigmoidal type III responses, which emerge when the searching rate coefficient (*a*) is an increasing function of prey density (Kalinkat et al., 2023; Uszko et al., 2020). Since θ > 1 can take any value, no single type III response exists. Prey searching rate (α) and, therefore, ingestion rate decreases overproportionately in type III when prey is scarce (Holling, 1959). Equation 4 was modified with θ = 2 to yield Holling’s type III FR (reviewed by Kalinkat et al., 2023; Okuyama, 2013; Okuyama and Ruyle, 2011):


(6) $$I=\frac{a\,P^{\text{2}}}{1+a\,h\,P^{\text{2}}}$$

Holling’s “disk” equations (Eq. 4−6) assume constant prey and predator levels, an oversimplification in many experiments, particularly with aquatic protists (discussed below, section 4.3). Whether there is also a type IV FR, with the ingestion rate declining at very high prey density, is a matter of debate (Bolker, 2008; DeLong, 2021).

Alternatively to Eq. 5, the type II FR can be expressed in the Michaelis–Menten form (Eq. 9 in Table 1). Equation 5 can be transformed into the Michaelis–Menten equation by multiplying the numerator and denominator by 1/*ah* (DeLong, 2021). In the figures shown in Table 1, α corresponds to the initial slope of the growth and ingestion curves. The maximum ingestion rate (*I*_*max*_) is equal to 1/*h*.

As I will point out using the empirical study reported in section 3.2, choosing the correct FR type is often difficult due to the inherent wide scattering of the experimental data in many studies (DeLong, 2021; Jeschke et al., 2004). As mentioned above, the parameters of the NR and FR equations are not constants but are affected by a suite of factors (e.g., habitat structure, changing prey and predator density during the experiment, nutritional history of prey and predator, temperature, etc.; DeLong, 2021; Kalinkat et al., 2023; Weisse et al., 2016a).

Grazing rates can be calculated from the change of prey numbers in experimental containers with predators relative to the shift of prey numbers in controls without predators (Eq. 8, Table 1). As I will discuss further below, this dependence of experimental results on changes occurring in independent controls is a significant issue for obtaining reliable results.

Clearance rate (*C*), i.e., the rate at which organisms process water, can be calculated from ingestion rate (*I*) and prey abundance (*P*) (Eq. 10, Table 1), with dimensions of volume per time (Fenchel, 1980; Fenchel, 1987). The clearance rate is equal to the proportion of prey consumed by the predator (Juliano, 2001; Trexler et al., 1988). The shape of the clearance rate curve depends on the type of functional response. It is instrumental in finding the most adequate FR model. In type I FR, *C* increases linearly up to a maximum and remains constant at further increasing prey density. In type II FR, *C* continuously declines, approaching the asymptote hyperbolically as prey density increases (Figure B in Table 1). In type III, *C* increases up to the inflection point of the sigmoid FR curve, and then decreases at further increasing prey density (DeLong, 2021; Juliano, 2001; Rothhaupt, 1990; Uszko et al., 2020).

Note that the clearance rate is not measured but derived from the measured ingestion rate (Eq. 7), causing issues of non-independence of parameter estimates (Berges, 1997; Montagnes, 2013; Uszko et al., 2020). A comparison of *C* across different taxa is only meaningful for the maximum clearance rate (*C*_*max*_) (Fenchel, 1987; Montagnes, 2013).

Combining NR and FR experiments yields further derived parameters such as gross growth efficiency (Eq. 11, Table 1).

## 2 Materials and methods

### 2.1 Organisms used and experimental design

I will illustrate the issues inherent in many NR and FR experiments using the planktonic freshwater ciliate *Pelagostrombidium mirabile* Penard, 1916; Krainer, 1991 (order Oligotrichida) as predator and the phototrophic flagellate *Cryptomonas* sp. as prey. *Pelagostrombidium mirabile* feeds primarily on algae but may also ingest small heterotrophic flagellates and bacteria (Macek et al., 2001). Ingested algae may serve as kleptoplasts, enabling the ciliate to perform mixotrophy when food is scarce (Stoecker et al., 2009).

The ciliate was isolated from the epilimnion of oligomesotrophic Lake Mondsee (Austria) in spring 2021. Clonal cultures were obtained from enrichment cultures using *Cryptomonas* sp. strain 26.80 provided by the Culture Collection of Algae in Göttingen (Germany) as food. The cultures used WC medium diluted with sterile-filtered lake water (FLW) collected from the hypolimnion of Lake Mondsee.

Ciliate stock cultures were kept in culture flasks (50 mL volume) with *Cryptomonas* sp. at constant light conditions (100 μmol photons m^–2^ s^–1^) and temperature (15°C). Experiments were conducted at low light levels (10−25 μmol photons m^–2^ s^–1^, 12:12 light:dark cycle) and a temperature of 10°C over food abundances ranging from < 1,000−70,000 *Cryptomonas* sp. cells mL^–1^. The low light level was chosen to discourage the algae from growing during the experiments.

The ciliates were gradually acclimated to the experimental temperature and prey levels over 5−7 d. The volume at each prey level was 20 mL during the acclimatization period to allow for adjusting target prey and predator levels. The final adaptation period at the experimental target food levels lasted 24 h.

The experimental conditions were non-axenic, but the bacterial background level checked by acoustic flow cytometry (Weisse et al., 2024) was low and bacterial ingestion was negligible (data not shown). The experiments were run in 6-well plates of 10-mL volume each for 24 h. The experimental design was similar to that used by Lu et al. (2021). Target prey density was adjusted by applying a geometric progression method, increasing prey abundance by a factor of 1.1–1.5 between each neighboring well, beginning with the lowest prey level. Note that, due to limitations imposed by using different volumes of media, prey and predator cultures when preparing the target levels (see Supporting Table S2A for details), it is virtually impossible to increase prey density in an ideal geometric progression.

Predator-free controls were run at approximately every second prey abundance as used in the experimental containers with the ciliate under identical conditions.

Subsamples for determining ciliate and food prey abundances were taken at the beginning (from the 20-mL containers of the final acclimatization stage used as inoculum) and end of the experiments (from each well) and immediately fixed with acid Lugol’s solution. Cell numbers of predators and prey were counted microscopically in sediment chambers of 3-mL volume (ciliates), respectively, in Sedgwick rafter cells of 1-mL volume (*Cryptomonas* sp.).

Ciliate growth rates were calculated from the change in cell numbers according to Equation 1 (Table 1). The geometric mean prey concentration (P) was determined using Eq. (2) (Table 1).

Ingestion rates (*I*) and grazing rates (*g*) of *Pelagostrombidium mirabile* were estimated from Eq. (7) and Eq. (8) (Table 1). The gross growth efficiency of *Pelagostrombidium mirabile* during the experiments was determined from Eq. (11) (Table 1).

Numerical and functional responses were calculated using the initial or the geometric mean prey concentration during the experiments.

Like in similar previous studies in our laboratory (e.g., Lu et al., 2021), the prey cell volume was measured by an electronic particle analyzer (CASY 1-model TTC; Schärfe System, Reutlingen, Germany). During the experiments, the average cell volume of *Cryptomonas* sp. was 221 μm^3^. The ciliate was sized alive by an imaging flow cytometer (FlowCam^®^, Fluid Imaging technology, Yarmouth, ME, USA; for details, see Weisse et al., 2023b).

The flagellate cell volume (in μm^3^) was converted to carbon units (pg C cell ^–1^) to express prey levels as biomass, assuming C = 0.261 × volume^0.860^ (Menden-Deuer and Lessard, 2000). Accordingly, an average *Cryptomonas* sp. cell contained 27 pg C.

The ciliate cell volume was estimated from length and width measurements, assuming the shape of a prolate ellipsoid. The ciliate’s average cell volume (63,650 μm^3^) was then converted to biomass (ng C cell^–1^) assuming C = 0.216 × volume^0.939^ (Menden-Deuer and Lessard, 2000), yielding 7.00 ng C ciliate^–1^.

### 2.2 Statistical analyses

Nonlinear curve fitting and graphics of the numerical (NR) and functional responses were performed in R (version 4.0.5; R Core Team, 2021) and SigmaPlot for Windows (version 14.5.0.101). The NR models used Eq. 3 to fit the initial or mean prey abundance (Eq. 2) during the experiments. Equations 4 and 9 were used to calculate type II and III FR models. Like the NR, the FR models used the initial or the mean experimental prey levels.

I first calculated the ingestion rates based upon the grazing rates (*g*) derived from changes in prey abundance occurring in the experimental containers and controls without predators (Eq. 8). Since at low prey levels, the experiments yielded biologically meaningless negative grazing rates, I repeated the analysis without considering changes of prey abundance in the controls (i.e., assuming *g* = μ [× − 1] in Supplementary Table S2B). Accordingly, the uncorrected ingestion was all positive. Thus, I could test if log-transforming the feeding rates improved the parameter fits. The results were similar for type II or type III responses. Therefore, I plotted the proportion of prey ingested vs. the initial prey abundance (i.e., the clearance rate) and fitted linear polynomial regressions to the data to differentiate between a type II or type III response. The quadratic model received the best support.

I used linear models to test whether the relative volume of filtered lake water (FLW) added to the containers and predator abundance (Rm, only in the experimental containers) affected the prey growth rates reported in Supplementary Table S2. Likewise, I tested if the factors FLW and Rm affected the ciliate’s ingestion rates.

The Akaike information criterion corrected for small sample size (AICc) was used for model selection [AICcmodavg package, (Mazerolle, 2023)].

The main body of text reports mean values with their standard error (SE) or standard deviation (SD). Results were considered significant if p was < 0.05.

## 3 Results

### 3.1 Prerequisites for the ideal experiment—experimental setup reducing the “noise”

Numerical and functional response experiments with phagotrophic aquatic protists are performed at many (> 10) food levels ranging from near-to-zero to satiating food conditions, usually reached at several tens of thousands of prey cells per mL. Different from the traditional approach, which investigated a certain number of fixed food abundances with several replicates each, selecting many food levels without replication is more appropriate to obtain an adequate nonlinear curve fit of the experimental results (Montagnes and Berges, 2004). In particular, the latter approach, which consists of many replicated experiments, often yields a better fit at low food levels and at the food concentrations at which growth and ingestion rates level off and allows for specification of the error terms in the parameter estimates (Montagnes and Berges, 2004). In contrast to prey levels, ideally, predator abundance should be identical in the individual experiments to avoid predator interference. In practice, this goal is difficult to meet due to the patchiness in the experimental bottles and the associated sampling error. Due to my experience, caution is needed if the predator abundance deviates by more than 20% among the experimental containers. The probability of predator interference should be tested statistically (see next section). Secondly, predator density must be high enough to allow precise estimates of changes in their cell numbers during the experiment. A minimum of 50 cells each, counted at the beginning and end of the experiment, usually meets this criterion.

In the following, I will use a hypothetical example to illustrate the steps of and difficulties inherent in designing and analyzing an “optimal” laboratory experiment. Setting up a series of NR or FR experiments with prey levels ranging from < 1,000 cells mL^–1^ to ∼100,000 cells mL^–1^ requires inoculating the experimental bottles with different volumes of the dense prey culture and medium (Supplementary Table S1), inevitably causing bias. In the theoretical example shown in Supplementary Table S1, the container volume is 50 mL, which has been used in many NR and FR experiments with small ciliates and dinoflagellates (Hansen, 1995; Gismervik, 2005; Weisse et al., 2021b). The experimental artifacts may be reduced if a part of the prey culture is gently filtered through fine mesh gauze to remove prey cells and then added to the experimental containers to ensure that a nearly identical amount of unfiltered culture volume (D_y2_ in Supplementary Table S1) plus filtered culture volume (FPC) is added to each experimental container. Similarly, an identical but filtered volume of predator culture (D_x__filt_, i.e., 3 mL in the example) should be added to each control. To complicate matters further, some protists, such as the well-known ciliates *Paramecium*, *Tetrahymena* and *Euplotes*, are sensitive to changes in their population density and social interactions (Weisse and Sonntag, 2016). These protists are adversely affected by any form of dilution, no matter whether prey culture, lake water (FLW) or medium is added. In such cases, the best strategy is to filter a part of the predator culture for dilution to obtain the experimental target levels (i.e., replacing FLW or FPC with D_x__filt_ in the experimental containers). For marine protists, filtered seawater can be used instead of FLW.

The above experimental design has several drawbacks. Firstly, using FPC instead of filtered lake water (FLW) requires a relatively large volume of the prey culture to set up the experiments (Σ [FPC + D_y2_], i.e., 1,128 mL in the example shown in Supplementary Table S1). Secondly, filtering the prey culture may not work with small prey (< 5 μm), which may squeeze through the mesh gauze. Bacterial prey can be separated from the predator using 0.8-μm or 1.0-μm filters for bacterivorous protists. Thirdly, since prey cultures are usually dense, the filters may clog, making large-volume filtration cumbersome. Note that sterile filtration should be avoided in non-axenic cultures because this would reduce the bacterial levels in the filtered prey cultures. I provide Supplementary Table S1 as a spreadsheet for setting up similar experiments.

In NR experiments, the predators’ specific growth rates (*r*) are calculated from changes in their cell numbers between final and initial samples (Eq. 1, Table 1). Predator growth rates are then related to either the initial food abundance or, to account for changes in prey levels during the experiments, the geometric mean food density (*P*) in each experimental bottle (Eq. 2). Cell numbers of prey often decline during the experiments, especially at low food levels, because the grazing effect of the predator is stronger than the growth rates (μ) of the prey. If the opposite holds, the initial prey density will underestimate the food available for the predator during the experiments.

However, prey levels often also change in controls that lack predators. For instance, food levels may decline if experiments with phototrophic prey are performed in the dark or under limiting nutrient conditions. More frequently, prey levels increase without predators if the inoculum is taken from exponentially growing cultures. Controls do not directly affect the calculation of the NR parameters. Still, they should be run parallel to the experimental bottles to rule out that prey levels decline for reasons other than the grazing effect of the predators (“bottle effects”; Weisse et al., 2021b). If negative prey growth rates are recorded, they should be more negative in the experimental containers than in the controls.

Unlike NR experiments, changes in prey abundance in controls directly affect the experimental outcome of FR experiments. This is because, by default, ingestion rates (*I*) are calculated from changes in prey cell numbers occurring in the experimental containers relative to those recorded in the control bottles (Eq. 7 and 8, Table 1). An inherent assumption is that prey growth is identical in experimental and control bottles, irrespective of the presence or absence of predators. However, as I will demonstrate in the next section, this premise is often violated, thus compromising the experimental results. Ideally, a control should be run at each experimental food level, but this is impractical because this would inflate the number of experimental and control bottles running parallel. Therefore, it is more appropriate to measure the change in prey cell numbers (i.e., prey growth rate, μ) in a lower number of controls and extrapolate the results for the respective experimental food densities. In the example shown in Supplementary Table S1, every second experimental prey density of the experimental series was used in the controls. This example assumes that the prey cells can be removed from the predators by filtration (D_x__filt_ in Supplementary Table S1). If this is not possible, the volume of the prey culture added (D_y2_) needs to be adjusted to yield the same target prey densities (P_ini_).

Not only because of the time needed to process many experimental treatments, the number of experimental and control containers cannot be increased *ad libitum*. In the theoretical example shown in Supplementary Table S1, the volume of each experimental container is 50 mL. Even without using the FPC approach, the experimental series shown in Supplementary Table S1 requires a minimum volume of the ciliate predator culture of 108 mL and 483 mL of the algal prey culture. However, it is often necessary to add predator and prey cells during the acclimatization period to keep the target densities constant, especially at the lower prey densities tested. Accordingly, a realistic scenario of the above experimental series would comprise approximately 150 mL of the dense ciliate culture and 600 mL of the prey culture. Strongly reducing the container volume is not an option because a minimum sample volume of 10 mL is required to accurately count the predator cell number at the beginning and end of this experiment. Since sampling error is inherent in subsampling, this causes inevitable noise of NR and FR experiments. The sampling error usually decreases with increasing sample volume, respectively, increasing prey and predator cell numbers counted.

In NR experiments, subsamples are taken to determine initial and final predator and prey numbers (R_0_ and R_24_, respectively, P_0_ and P_24_; Supplementary Table S2). The sampling error is twofold if predator and prey numbers can be measured in the same sample. However, at high food densities, it may be necessary to count predator and prey abundance in different subsamples because high cell numbers of prey may mask some predator cells, resulting in an underestimation of predator abundance. The disadvantage of this strategy is that the sampling error then is fourfold.

Provided that predator and prey abundance can be assessed in the same sample, a fourfold sampling error is the default in FR experiments because initial and final cell numbers need to be counted in experimental and control bottles.

With all those limitations and potential pitfalls in mind, how does this translate into setting up and analyzing a “real” experiment?

### 3.2 Illustrating the practical issues—an example from a “problematic” laboratory experiment

The range of target prey levels (P_ini_) in the experimental containers with the ciliate *Pelagostrombidium mirabile* and controls without the predator (Supplementary Table 2A) was similar to the theoretical example in Supplementary Table 1. However, the experimental volume was smaller than in the previous example. Since the flagellate *Cryptomonas* sp. is small and slender (∼10 × 4μm), effectively removing the prey from the predator by filtration did not yield satisfactory results. Accordingly, control bottles were inoculated from ciliate-free cultures. The measured initial prey density (P_0_) deviated from the respective target levels at higher prey abundances. The average ratio P_0_/P_ini_ was 0.65 ± 0.21 (SD) in the experimental containers and 0.72 ± 0.17 in the controls (Supplementary Table 2A). In contrast, the initial predator abundance did not deviate significantly from their target levels (*R_0_/R_*ini*_* = 1.10 ± 0.12).

The experimental results are reported in Supplementary Table 2B. Unexpectedly, the prey levels declined not only in the experimental containers but declined even stronger in the predator-free controls. In the controls, the percentage of filtered lake water (FLW) added to the containers was higher (70–99.5%) than in the experimental containers (45–90%, Supplementary Table 2A). Therefore, I tested if the relative volume of FLW added to the containers affected the flagellate’s growth rates. In the controls, *Cryptomonas* growth rates declined linearly with increasing FLW volume (*p* = 0.029). In the experimental containers, the negative FLW effect was also significant (*p* = 0.020). In contrast, the predator abundance (Rm in Supplementary Table S2B) did not affect (*p* = 0.829) *Cryptomonas* growth rates.

**FIGURE 1:**
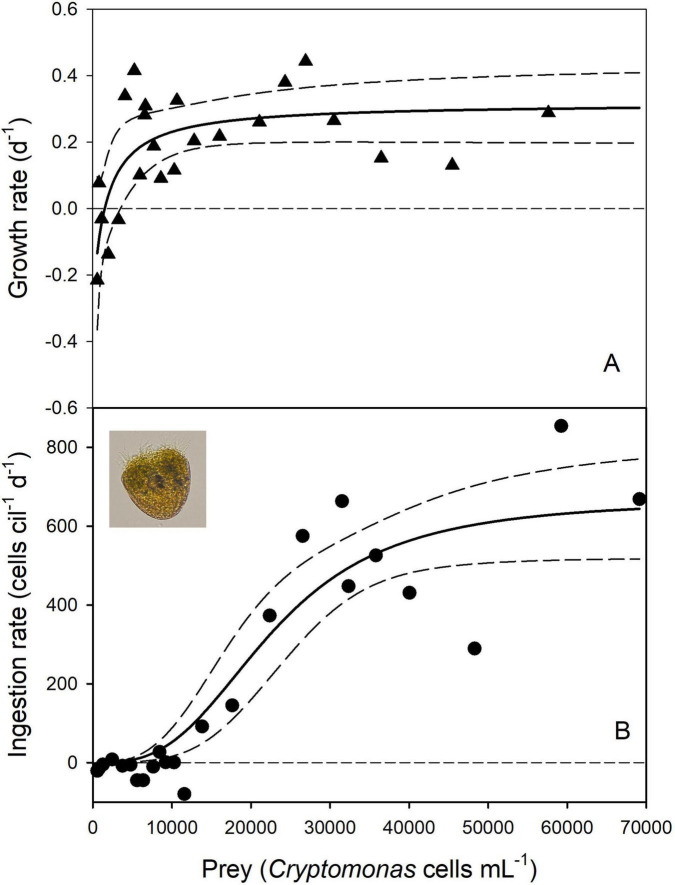
Numerical response **(A)** and type III functional response (with the Hill exponent θ = 3) **(B)** of the ciliate *Pelagostrombidium mirabile*. The solid lines show the non-linear regressions and the dashed lines indicate the 95% confidence bands. The inset in **(B)** shows a Lugol’s fixed ciliate cell. Prey density is the mean abundance **(A)** or the initial abundance at the beginning of the experiments **(B)**.

Fitting the NR equation (Eq. 3) to the experimental data yielded a reasonable curve fit (Figure 1A) and significant estimates for the maximum specific growth rate (*r*_*max*_ = 0.320 ± 0.054 d^–1^, *p* < 0.0001) and the threshold prey density (*P*′ = 1,349 ± 479 cells mL^–1^, p = 0.0097; Supplementary Table S3A). If expressed in units of carbon per liter, the threshold level was 0.036 mg C L^–1^. The estimate of the constant *k*_2_ was not significant (p = 0.165). Food saturation was reached at approximately 40,000 prey cells mL^–1^, corresponding to prey biomass of 1.08 mg C L^–1^.

A linear model indicated that the FLW addition and Rm negatively affected the ciliate’s ingestion rate, indicating some predator interference (*p* < 0.001 for each factor).

The above parameter estimates were derived by using the geometric mean prey abundance during the experiments. Replacing the mean prey density with the initial prey abundance yielded results that were not significantly different (Supplementary Table S3B). Note that more negative AICc scores suggest a better model fit. However, the difference was well below the critical ΔAIC of 2 (Burnham and Anderson, 2004). Accordingly, both models have substantial support and can be used to fit the NR of *P. mirabile*.

The declining *Cryptomonas* sp. levels in the controls during the experiments strongly affected the parameter estimates of the ciliate’s functional response using equations 4, 7, and 8. At initial prey densities (P_0_) < 20.000 cells mL^–1^, the flagellate growth rates were more negative in the controls than in the experimental containers (Supplementary Table S2B), resulting in biologically meaningless negative feeding rates (*g* and *I* in Supplementary Table S2B). Due to the wide scattering of prey growth rates, interpolating from changes of *Cryptomonas* cell numbers in the controls (μ) to each prey level in the experimental containers for calculating *g* (using Eq. 8) was impossible. Therefore, *Cryptomonas* growth rates in the controls averaged over several prey densities (indicated by the color code in Supplementary Table S2B) were used to calculate *g*.

Positive ingestion rates were obtained at mean prey levels (P_m_) exceeding ∼10,000 cells mL^–1^. A sigmoidal type III FR model with the Hill exponent θ = 3 and the initial prey densities yielded the best support (Figure 1B and Supplementary Table S4A). The estimates of the space clearance rate *a* and the handling time *h* were significant; the AICc score was 231.5 (Supplementary Table S4A). The type III model that replaced the initial by the mean prey levels received significantly less support (AICc = 238.3, Supplementary Table S4B). However, both models yielded the same *h*, and because *I*_*max*_ is 1/h, they have identical estimates of *I*_*max*_ (667 prey cells ciliate^–1^ d^–1^).

**FIGURE 2:**
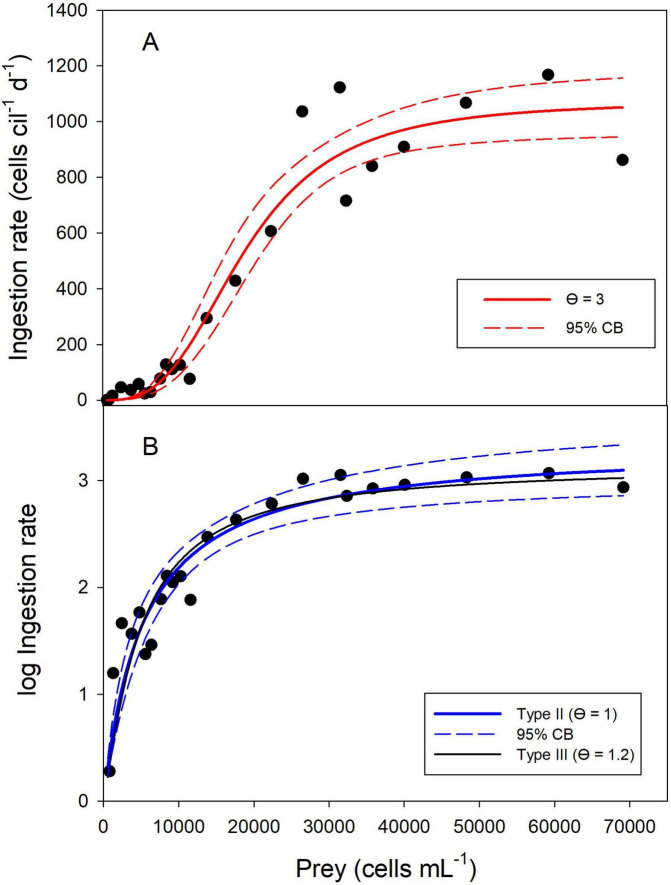
Functional response of the ciliate *P. mirabile* without correcting for changes in food levels in the controls. A type III FR model with θ = 3 yielded the best fit for untransformed ingestion rates **(A)**. After logarithmic transformation, a type II FR and a slightly sigmoidal type III FR (θ = 1.2) received similar support **(B)**. The solid lines show the non-linear regressions and the dashed lines indicate the 95% confidence bands. Prey density is the initial abundance at the beginning of the experiments.

If the changes of the prey densities in the controls were ignored and the initial prey densities were used for the curve fitting, the best type-III model (with θ = 3) estimated a lower handling time and, therefore, a higher *I*_*max*_ (1,073 prey cells ciliate^–1^ d^–1^, Supplementary Table S5A and Figure 2A) than the above models using the conventional calculation of *g* (Eq. 8, Table 1). Further increasing the hill exponent (up to θ = 3.5) did not receive better statistical support. If θ was set to 2, the default parameter of Holling’s type III, the AICc score was significantly higher (AICc = 236.9, Supplementary Table S5B) than in the previous model.

Because the grazing coefficient was always positive if the uncorrected dataset was used for calculating the ingestion rates, I could test if logarithmic transformation improved the curve fit. This was seemingly the case (Figure 2B), with a surprising result: the type II FR yielded the best model fit (Supplementary Table S6A) but predicted *I*_*max*_ (2,143 prey cells ciliate^–1^ d^–1^) distinctly higher than the measured values shown in Figure 2A. Slightly sigmoidal type III models (with θ = 1.1 and θ = 1.2) yielded model fits that were visually (Figure 2B) and statistically (Supplementary Tables S6B,C) virtually indistinguishable from the type II FR. The type III model with θ = 1.1 was not statistically different from the type II model; the ΔAICc score of the type III model, assuming θ = 1.2, was 2.17 higher than the type II FR. However, the estimated *I*_*max*_ (1,392 prey cells ciliate^–1^ d^–1^) of the latter better matched the measured values than the type II model. In short, after log-transformation, it was impossible to conclude if the ciliate’s functional response followed a rectangular hyperbolic type II or a slightly sigmoidal type III response.

**FIGURE 3:**
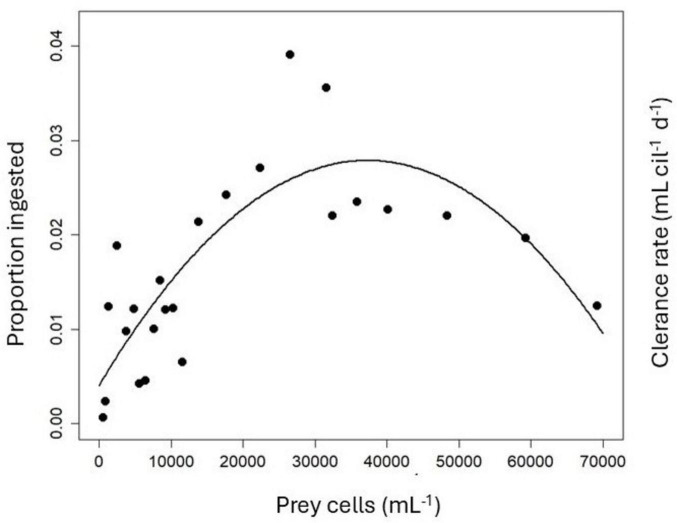
Proportion of prey ingested (= Clearance rate) by *P. mirabile* vs. initial prey abundance without correcting for changes in food levels in the controls.

To differentiate clearly between a type II or type III response, I plotted the proportion of prey ingested (i.e., the clearance rate, Eq. 10) vs. the initial prey abundance and fitted polynomial regressions to the data (Figure 3). A second-order linear model yielded a significantly linear term (*p* = 0.0016) and a significantly negative quadratic term (*p* < 0.0001), conforming to a type III response (DeLong, 2021). The maximum clearance rate measured was 39.1 μL cil^–1^ d^–1^, corresponding to 3.9% of the available prey ingested (Figure 3).

The above estimates of *I*_*max*_ obtained for *P. mirabile* differed widely, depending on whether or not the ciliate’s feeding rates were corrected for changes in prey densities occurring in the controls. I calculated the gross growth efficiency (GGE, Eq. 11 in Table 1) to obtain evidence which may be the more likely scenario. Assuming *r*_*max*_ = 0.32 d^–1^, *I*_*max*_ = 667 prey cells ciliate^–1^ d^–1^, the mean prey volume of 221 μm^3^, and the average predator cell volume of 63,650 μm^3^, the GGE was 0.14. If we assume the higher *I*_*max*_ (1,073 prey cells ciliate^–1^ d^–1^) obtained by the type III FR model using the uncorrected dataset, the GGE was 0.09. In terms of cell carbon, the corresponding GGEs were 0.12 and 0.08.

## 4 Discussion

### 4.1 Evaluating the experimental results

The parameter estimates from an NR or FR study must be critically evaluated to avoid reporting biased results. This should comprise (*i*) a comparison with published previous work and (*ii*) a check for internal consistency of the findings obtained.

(ad *i*) The food threshold, the saturating food biomass, the specific growth rate, and the range of the maximum ingestion and clearance rates reported in this study for *Pelagostrombidium mirabile* all fall within the range typical of algivorous ciliates (Gismervik, 2005; Kivi and Setälä, 1995; Weisse, 2006) and references therein). For the same species and a temperature of 15°C but under otherwise similar experimental conditions, Weisse et al. (2023a) reported a food threshold of 0.15 mg C L^–1^, food saturation at ∼2 mg C L^–1^, and *r*_*max*_ of 0.58 d^–1^. These results are comparable to the numerical and temperature responses reported for marine and freshwater oligotrich and other ciliates (Gismervik, 2005; Lu et al., 2021; Lukić et al., 2022; Montagnes, 2013). I conclude that the NR models yielded valid parameter estimates for *P. mirabile* at both temperatures investigated [i.e., 15°C (Weisse et al., 2023a) and 10°C (this study)].

(ad *ii*) The most significant issue affecting the functional response parameters was the negative prey growth rates in the controls, which declined more than in the experimental containers. The best practice for calculating the grazing rate *g* (Eq. 8, Supplementary Table S2B) was somewhat subjective. More importantly, the addition of FLW, which was higher in the controls, adversely affected the flagellate growth rates. I assume that dilution with FLW reduced nutrients and vitamins in the wells. The nutrient levels in the prey and predator cultures and the FLW were not measured before the beginning of the experiments. However, the dilution may not fully explain why high additions of FLW yielded negative feeding rates. Therefore, I did not publish this case study earlier. However, in the present context, this work is instrumental in demonstrating the manifold pitfalls inherent in many FR experiments. To get a handle on the effect of the bias originating from the FLW addition, I presented the experimental FR results with and without correcting the measured prey growth rates in the experimental containers for changes in cell numbers occurring in the controls. The different models indicate a range of parameter estimates (i.e., *I*_*max*_, *C*_*max*_, α, *h*) that can be compared to the literature.

The gross growth efficiency (GGE), i.e., the fraction of prey biomass converted into predator biomass (Table 1), can only vary between 0 and 1 and, therefore, provides a check for consistency between NR and FR studies (Gismervik, 2005). The estimated GGE of *P. mirabile* ranged from 0.08 to 0.14, depending on the method used. This is at the lower end of the GGE of ciliates and other aquatic protists (Straile, 1997) but similar to recent estimates obtained with two other freshwater ciliates under comparable experimental conditions (i.e., identical food, temperature, and experimental design; Lu et al., 2021). Comparably low GGEs are also known from marine ciliates (Gismervik, 2005; Straile, 1997).

The GGE of ciliates and other taxa is generally affected by temperature (Straile, 1997). Notably, Lu et al. (2021) reported the lowest GGE for both ciliates studied at 10°C; the experimental temperature ranged from 5 to 20°C in their study. I did not investigate if the temperature also affects the GGE of *P. mirabile.* Concerning the maximum ingestion rates, I conclude that it is impossible to decide which FR scenarios (i.e., the FR models using either the corrected or the uncorrected dataset) are more likely.

Despite the uncertainty arising from the more negative prey growth rates in the controls than in the experimental wells, the various FR models reported above suggest that *P. mirabile* follows the rare sigmoidal type III FR (Gismervik, 2005; Kalinkat et al., 2023). If several food resources are available, prey switching to a better food source is the conventional explanation for measuring a type III response (Kalinkat et al., 2023). However, this does not apply to the present study’s single-resource scenario. Similarly, spatial refuges allowing the prey to escape predation in a spatially complex habitat (Kalinkat et al., 2023) appear unlikely for the present study. For ciliates and other aquatic protists, the most likely explanation for a type III FR is a decrease in searching (or attack) rate when prey are scarce, presumably to reduce energy expenditure (Montagnes, 2013; Weisse et al., 2016a). The low GGE measured for *P. mirabile* feeding on *Cryptomonas* sp. may suggest that the flagellate was suboptimal food for the ciliate. Accordingly, the energy gain might have become negative at low prey density. Therefore, suboptimal resources may lead to type III FR (Kalinkat et al., 2023).

I will discuss in the next section that issues arising from the acclimatization to the experimental conditions and different temperature sensitivity of prey and predator may also have adversely affected the experimental outcome in the present study.

### 4.2 Advantages and drawbacks of acclimatization

A common goal in experimental research is to standardize the conditions to obtain reliable and reproducible results. To this end, the need to precondition protists to the experimental conditions has been stressed repeatedly (Beveridge et al., 2010; Franzè and Menden-Deuer, 2020; Li et al., 2013; Meunier et al., 2012; Montagnes and Franklin, 2001). The nutritional history (i.e., past–prey availability and nutritional quality of the prey) affects the predators’ numerical and functional responses (Boenigk et al., 2001; Calbet et al., 2013; Li et al., 2013). Similarly, rapid changes in abiotic factors (e.g., temperature, pH) are often detrimental to both predator and prey (Franzè and Menden-Deuer, 2020). Therefore, protists need to be acclimatized gradually to the experimental conditions.

There is some confusion in the literature about the terms acclimatization, acclimation and adaptation. I use the term “acclimatization” instead of the often used “acclimation” (e.g., Franzè and Menden-Deuer, 2020; Lu et al., 2021) to describe the reversible and temporary adaptation process of living organisms to a changing environment (i.e., in this context, the experimental conditions) within a short time (days). This conceptually differs from adaptation, a gradual, long-term and irreversible process shown by living organisms over many generations to adjust to a new environment or changing habitat, such as an increase in temperature. After adaptation, organisms are acclimated to, e.g., new climatic conditions.

As a rule of thumb, the acclimatization period should last at least for one generation (ideally, three generations for herbivorous species; Franzè and Menden-Deuer, 2020) of the study protist for each stepwise change of culture conditions. This premise is difficult to achieve at the edge of the tolerance range of a given protist species. For instance, growth rates usually decline overproportionally at the minimum and maximum temperature tolerated, increasing the length of the generation time and, therefore, the period of acclimatization. Under such suboptimal conditions, protists may no longer grow exponentially. The final adaptation period (24 h) used in the experiments with *Pelagostrombidium mirabile* (this work) was equivalent to only half its generation time G (G = 2.17 d or 52 h, since G = ln 2/*r*_*max*_) and, therefore, probably too short.

Standardization of the preconditioning is further complicated if prey and predator have distinctly different temperature optima and pessima. Suppose the prey is more sensitive than the predator is to changing temperature. In that case, this may require that food is replenished repeatedly (i.e., more often than close to its optimum temperature) during the acclimatization period to maintain the experimental target food level. This was the case in the experimental study reported above because the lower temperature tolerance of *Cryptomonas* sp. is close to 10°C (Weisse et al., 2016b; Wirth et al., 2019). However, adding food without diluting the predator abundance is nearly impossible. Therefore, it is difficult to maintain similar predator abundances and nutritional quality of the prey across all experimental food levels during the acclimatization period. In the vicinity of the temperature optimum, protist numbers will increase more rapidly than at the lower and upper temperature limits, requiring smaller inocula to the experimental containers than at suboptimal temperatures. As outlined above, the different inocula volumes of predator, prey, medium, and FLW in the experimental containers inevitably cause different initial conditions at the various food levels used in NR and FR experiments. However, this drawback appears minor relative to the bias introduced by refraining from preconditioning the protists to the experimental conditions.

### 4.3 Statistical issues affecting the numerical and functional responses

Providing identical experimental conditions at a given prey level with and without predators is virtually impossible. Firstly, especially in fast swimming protists such as ciliates and dinoflagellates, a small amount of prey is nearly always introduced to the experimental containers together with the predators, and this prey may physiologically (i.e., stoichiometrically or nutritionally; Meunier et al., 2012) deviate from those cells used to provide the target food abundance at the various experimental levels. Secondly, most experiments investigating the FR of omnivorous, herbivorous or bacterivorous protists are not monoxenic but contain an often-unclassified bacterial background flora accompanying the target prey. For instance, offering suspended algae axenically to an algivorous (or omnivorous) predator in NR and FR experiments is difficult. The bacterial cell numbers can be counted in small subsamples (∼1 mL) by flow cytometry or epifluorescence microscopy, and the potential bacterial uptake by the protists can be accounted for Chen et al. (2020); Weisse et al. (2021a). However, the bacterial background flora may be quantitatively and qualitatively different in the inocula with and without protist predators. Furthermore, bacterial growth may be stimulated by the activity of protists (i.e., via direct or indirect nutrient release by excretion, cell death and sloppy feeding) in the experimental containers, thus violating the assumption that the bacteria behave identically in experimental and control flasks. At low nutrient levels, nutrient release by heterotrophic protists may also enhance the growth rates of phototrophic prey and bacteria in the experimental containers relative to controls without predators.

Because the calculation of grazing and ingestion rates are sensitive to changes in prey cell numbers in both experimental containers and controls, the noise is usually more prominent in FR than in NR experiments. However, it is not only higher stochasticity but also bias that generally causes a more extensive scattering of experimental results in FR experiments than in NR experiments.

I used the general form of Holling’s disk equations to estimate the functional response parameters of the ciliate *Pelagostrombidium mirabile*. Like the numerical response (Eq. 3), Holling’s type I−III functional responses assume constant prey and predator levels. To account for the usually declining prey abundance in the course of an experiment, most microbial ecologists dealing with aquatic protists do not use the initial but the mean prey levels over the incubation (Eq. 2) to calculate the NR and FR parameters (Frost, 1972; Gismervik, 2005; Montagnes, 1996; Weisse et al., 2002). This correction also applies to the Michaelis-Menten form of the FR equation (Eq. 9). Alternatively, the Rogers Random Predator (RRP) equation and the Lambert Random Predator Equation (LRP) can be used to account for depleting prey levels (reviewed by DeLong, 2021).

Like prey abundance, predator abundance is not constant in NR and FR experiments as performed in this study. The predator abundance declines below the threshold prey density (*P*′, Eq. 3). If *P* > *P*′, the predator population increases up to the satiating prey density, at which *r*_*max*_ is reached (Figure A in Table 1). Predator interference may occur at high predator levels, reducing the *per capita* ingestion rate (Arditi and Ginzburg, 2012; DeLong and Vasseur, 2011; DeLong and Vasseur, 2013; Skalski and Gilliam, 2001). Therefore, even if the prey density is kept constant (Hewett, 1980), the ratio between prey and predator may change during an experiment, thus violating the assumptions of the standard type II FR (DeLong, 2021). Predator interference likely affected the ciliate’s ingestion rate, as reported in section 3.2, although the mean ciliate abundances varied relatively little at prey levels > 3,000 *Cryptomonas* cells mL^–1^ (Supplementary Table S2B).

Relative to the primary question of whether or not to correct the grazing rates for changes in prey levels occurring in the controls, the effect of the various modifications of the parameter estimates is minor. At least for type II, it seems clear that all of the above fitting approaches reliably estimate the FR parameters (DeLong, 2021).

Uszko and colleagues (Uszko et al., 2020) identified three major problems in FR analyses: (*i*) ill-chosen experimental prey densities that do not provide good coverage at low prey density, (*ii*) unevenly distributed variance of ingestion rates, and (*iii*) non-independence of the model parameters *a*, θ, and *h*. These authors demonstrated, theoretically and graphically, that an increase in the attack rate *a* similarly increases the height of the FR curve at low prey density as a decrease in the Hill exponent θ and, to a lesser extent, lowers the handling time *h.* However, θ mainly affects the sigmoidal aspect of the curve at moderate prey density and *h* the asymptote reached at satiating food levels (DeLong, 2021).

Importantly, different combinations of the three above parameters may yield similar FR curves (DeLong, 2021; Uszko et al., 2020). These authors further point out that the variance of ingestion rates increases with prey density. Uszko et al. (2020) recommend log-transforming the ingestion rate to remove heteroscedasticity and improve the accuracy and precision of the parameter estimates. However, the above laboratory experiment with *Pelagostrombidium mirabile* showed that log-transformation can erroneously lead to the assumption of the more common type II FR. Based on the information criterion, the latter received the best support. Still, the ΔAICc to the slightly sigmoidal type III FR was too small (0.85, Supplementary Table S6) to decide which of the two models was more likely. Plotting the proportion of prey ingested vs. prey density identified the ciliate’s sigmoidal functional response. The ciliate’s type III response became evident in each model, irrespective of assuming the initial or mean prey density and correcting or not the grazing parameter *g* for changes of prey density occurring in the controls.

Finally, individual variation is an essential driver of the stochastic predator-prey encounters (DeLong, 2021), affecting both NR and FR experiments. Both responses describe *per capita* rates of the predator as a function of prey density. Even if clonal protist populations are used, as in the case study presented above, there is individual variation in growth and feeding rates (Boenigk et al., 2007; Weisse and Rammer, 2006; Yang et al., 2013). If the abundance is kept constant, the effect of the intraspecific variation may decline with increasing container volume, but “bottle effects” may also decline with increasing experimental volume. A recent study demonstrated that container volume affected the growth rates of three of the five studied freshwater ciliate species (Weisse et al., 2021b). The predator population size was comparatively small (∼ 250 individuals), both in the theoretical example assuming a larger container volume (50 mL) and a lower predator density (∼5 mL^–1^) and in the laboratory experiment using small wells (10 mL) and a higher predator density (∼25 mL^–1^). However, chances for pronounced individual variation are higher if the population size is larger, say, 10,000 individuals (for instance, using experimental containers of 0.5 L volume and a predator density of 20 mL^–1^). Larger container volumes than 10 mL are needed if numerical and functional responses are assessed in multispecies experiments (see next section).

### 4.4 More practical issues, single resource vs. multispecies functional response, and implications for the food web

From the foregoing, it is evident that providing identical initial conditions apart from different prey abundances is principally impossible in NR and FR experiments with phagotrophic aquatic protists. The goal, therefore, is (*i*) providing quasi–identical initial conditions and (*ii*) staying close to the initial conditions in all containers. If the latter premise is not met and, e.g., prey cell numbers decline strongly during the experiment, growth and ingestion rates may be assigned to a geometric mean food concentration that was not typical of the experiment. However, the endeavor of maintaining the initial conditions conflicts with obtaining statistically reliable results for protist growth and grazing rates. Results calculated from changes in prey and predator cell numbers in experimental containers and controls become seemingly more accurate with increasing experimental duration. Yet, this is only the case if growth and grazing rates remain constant throughout the experiment.

The equations used to calculate growth and ingestion rates assume an exponential change in cell numbers of prey and predator, which is an assumption that may be violated under suboptimal conditions (see the previous sections). Even after careful acclimatization, a lag phase may occur due to the experimental manipulation. Such a lag phase cannot be detected if results are calculated from the initial and final protist abundance, as it is common practice in NR and FR experiments. The result is potentially underestimating the actual growth and ingestion rates. Accordingly, we are confronted with a trade-off between staying close to the initial conditions (i.e., favoring short experimental duration) and reducing the effect of the lag phase (i.e., favoring longer experimental duration) on the experimental results. The impact of the lag phase may be reduced if the initial samples (= start of the experiments) are taken ∼2–3 h after setting up the experiments (Weisse and Moser, 2020).

Most published NR and FR experiments with aquatic protists were conducted over 24 h. This duration is (*i*) close to the generation time of many marine and freshwater protists (Banse, 1982; Fenchel, 1987; McManus and Santoferrara, 2013; Weisse, 2006) and (*ii*) allows to neglect possible diurnal changes in growth and, for heterotrophs, feeding activities that are known from many phototrophic and some heterotrophic protist species (Armengol et al., 2019; Jakobsen and Strom, 2004; Ng et al., 2017).

The specific growth rates of a bacterivorous ciliate measured over 24 h were not different if the NR curves were fitted to the initial bacterial abundance or the mean bacterial biomass (Weisse et al., 2024). The authors concluded that if the respective food levels do not change enormously during the incubation, either method should yield reliable results. This conclusion is supported by the present work.

In addition to the above restrictions applying to all modifications of NR and FR experiments, enclosure experiments in general and small-scale laboratory experiments, in particular, can only partially mimic the natural conditions in the field (reviewed by Altermatt et al., 2015; Weisse et al., 2016a; Weisse et al., 2021b). In most cases, experimental standardization refers to one or, at best, a few selected biotic and abiotic factors, neglecting other variables and their interactions. For the growth of phototrophic protists, temperature × nutrient interaction and light × temperature interaction have been extensively studied (reviewed by Edwards et al., 2016; Thomas et al., 2017). The interactive effect of prey abundance, irradiance and pH has been studied for the NR and FR of the marine obligate mixotrophic ciliate *Mesodinium rubrum* (Smith and Hansen, 2007) and the closely related, non-symbiotic species *M. pulex* (Tarangkoon and Hansen, 2011). Similarly, the interactive effect of food and temperature has been demonstrated for the NR of several freshwater ciliates (Weisse et al., 2002) and the heterotrophic marine flagellate *Oxyrrhis marina* (Kimmance et al., 2006).

However, compared to phytoplankton, the study of interactive effects of biotic and abiotic factors on NR and FR of heterotrophic aquatic protists is still in its infancy. The most severe restriction probably is that the current knowledge is almost exclusively based upon single prey/single predator experiments, ignoring interactions with other biota that are always present in the natural environment. Even if NR and FR experiments tested several prey species, those were usually offered individually to the predator, with a few exceptions (Fox, 2007; Hewett, 1980). There is an urgent need to study and model the numerical and functional responses in multispecies experiments in general (DeLong, 2021; Koen-Alonso, 2007) and for aquatic protists, in particular. Prey switching can only occur if more than one food source is available. It seems, therefore, plausible that feeding of a given (protist) predator, which follows a type II FR in single resource experiments, switches to a type III FR in multispecies experiments. If prey switching is common among single keystone predators or several coexisting predators, this has significant implications at the ecosystem level. Prey that was preferred at high density may have a strongly reduced risk of predation at low prey levels. Therefore, different from the type II FR, type III FR tend to stabilize consumer-resource population dynamics and promote ecosystem biodiversity (reviewed by DeLong, 2021; Kalinkat et al., 2023).

## 5 Conclusion

In the foregoing, I have discussed why it is principally impossible to perform an “ideal” NR and FR experiment, fulfilling all criteria of experimental standardization. This is especially true of functional response experiments. Essentially, an FR experiment represents a series of individual experiments conducted in parallel to each other. Accordingly, the noise is often more significant than expected from a single multi-level experiment. Ecologists working experimentally with protists need to be aware of the various sources of error. They should pay the utmost attention to keeping the inevitable bias to an acceptable minimum. This goal is usually reached if the nonlinear statistical analyses used for calculating the experimental results provide significant goodness of fit of the NR and FR curves and significant parameter estimates. Importantly, maximum growth and ingestion rates predicted by the respective equations are only meaningful if they are reached at realistic food abundances, i.e., at food levels that are met in the natural realm (Weisse et al., 2024). Finally, the manifold practical and theoretical issues discussed in this work should not discourage researchers from investigating multispecies functional and numerical responses.

### 5.1 Recommendations for NR and FR experiments

• Use material from healthy, exponentially growing stock cultures.
• Gradually acclimatize prey and predator organisms to the experimental conditions.
• The acclimatization period should correspond to one or two generations, ideally at each experimental target level.
• To dilute dense cultures in experiments with robust species, use medium or filtered natural water with similar (ideally, identical) nutrient levels as the medium/natural water used.
• For predator species sensitive to dilution, use filtered predator culture for dilution.
• Make sure that predator abundance is similar (± 20% at the most) across all food levels, and, using appropriate statistics, test for potential predator interference.
• Check for contamination with non-target organisms (e.g., bacteria) and, if necessary,
• use mesh gauze or gentle filtration to remove contaminants (high-pressure filtration may increase resource levels such as amino acids in the filtrate). Adding antibiotics to the medium can affect the health of both the predator and the prey (e.g., Chen et al., 2020).
• Incubate all experimental and control containers randomly during the acclimatization and experimental period.
• Report the mean and range of abiotic conditions applied (e.g., temperature, light, pH).
• Do not take initial samples immediately after setting up the experiment, but wait for ∼2-3 h to reduce the potential effect of a lag phase.
• Run the experiments over a period corresponding to ∼1 generation time of the predator or, at least, 24 h.
• Count at least 50 cells of each prey and predator in each (sub)sample.
• Whenever possible, measure and report the mean and range of cell sizes of at least 30 cells of prey and predators each.
• Report factors used to convert cell volume to biomass.
• Use adequate statistical tests (available, e.g., in several packages provided by the free statistical programming language R) to obtain the best curve fit and evaluate the significance level of all parameters provided by the NR and FR equations.
• Report goodness of fit, confidence intervals, standard error or standard deviation and significance levels of all parameters from NR and FR curves.
• Select the model with the best statistical support based on information criteria.
• Make all experimental data freely available to other researchers.

## Data Availability

The original contributions presented in this study are included in this article/Supplementary material, further inquiries can be directed to the corresponding author.
