# Supplementary material for: Numerical and functional response of phagotrophic aquatic protists: the ideal experiment—and why we cannot get it
Source: Front Microbiol. 2025 Jun 10;16:1559802. doi: 10.3389/fmicb.2025.1559802 (PMC12186456; doi:10.3389/fmicb.2025.1559802)
Supplement: Supplementary file 3 [file Table_3.docx]

Table S3A. Results of the numerical response fit using equation (3) and mean prey abundances (see Table 1 and main text for details).

| Coefficient | Value | SE | t | p |
| --- | --- | --- | --- | --- |
| *r_max_* | 0.320 | 0.054 | 5.921 | <0.0001 |
| *P_0_* | 1,349 | 479 | 2.821 | 0.0097 |
| *k_2_* | 2,591 | 1807 | 1.434 | 0.165 |

AICc = -86.08

Table S3B. Results of the numerical response fit using equation (3) and initial prey abundances (see Table 1 and main text for details).

| Coefficient | Value | SE | t | p |
| --- | --- | --- | --- | --- |
| *r_max_* | 0.318 | 0.061 | 5.220 | <0.0001 |
| *P_0_* | 1,505 | 621 | 2.423 | 0.025 |
| *k_2_* | 3,192 | 2388 | 1.337 | 0.196 |

AICc = -85.75

Table S4A. Results of the functional response type III model fit using equation (1) and initial prey abundances. The Hill exponent (ϴ) was 3 (see Table 1 and main text for details).

| Parameter | Value | SE | t | p |
| --- | --- | --- | --- | --- |
| a | 5.64E-11 | 2.07E-11 | 2.719 | 0.0125 |
| h | 0.0015 | 0.0012 | 9.419 | <0.0001 |
| I_max_ (1/h) | 666.7 |  |  |  |

AICc = 231.49

Table S4B. Results of the functional response type III model fit using equation (1) and mean prey abundances. The Hill exponent (ϴ) was 3 (see Table 1 and main text for details).

| Parameter | Value | SE | t | p |
| --- | --- | --- | --- | --- |
| a | 1.15E-10 | 5.77E-11 | 2.622 | 0.156 |
| h | 0.0015 | 0.0002 | 8.734 | <0.0001 |
| I_max_ (1/h) | 666.7 |  |  |  |

AICc = 238.29

Table S5A. Results of the functional response type III model fit using initial prey abundances and grazing coefficients (g) that were not corrected for changes in prey cell numbers in controls. The Hill exponent (ϴ) was 3 (see Table 1 and main text for details).

| Parameter | Value | SE | t | p |
| --- | --- | --- | --- | --- |
| a | 1.59E-10 | 3.37E-11 | 4.714 | 0.0001 |
| h | 0.0009 | 4.83E-5 | 19.306 | <0.0001 |
| I_max_ (1/h) | 1073 |  |  |  |

AICc = 229.1

Table S5B. Results of the functional response type III model fit using initial prey abundances and grazing coefficients (g) that were not corrected for changes in prey cell numbers in controls. The Hill exponent (ϴ) was 2 (see Table 1 and main text for details).

| Parameter | Value | SE | t | p |
| --- | --- | --- | --- | --- |
| a | 2.51E-06 | 4.94E-07 | 5.072 | <0.0001 |
| h | 8.114E-04 | 6.70E-05 | 12.105 | <0.0001 |
| I_max_ (1/h) | 1232 |  |  |  |

AICc = 236.9

## Table S6A. Results of the functional response type II model fit using log-initial prey abundances and grazing coefficients (g) that were not corrected for changes in prey cell numbers in controls. The Hill exponent (ϴ) was 1 (see Table 1 and main text for details).

| Parameter | Value | SE | t | p |
| --- | --- | --- | --- | --- |
| a | 0.0006 | 8.90E-05 | 7.128 | <0.0001 |
| h | 0.3002 | 0.0141 | 21.342 | <0.0001 |
| I_max_ (1/h) | 2143 |  |  |  |

AICc = -54.34

## Table S6B. Results of the functional response type II model fit using log-initial prey abundances and grazing coefficients (g) that were not corrected for changes in prey cell numbers in controls. The Hill exponent (ϴ) was 1.1 (see Table 1 and main text for details).

| Parameter | Value | SE | t | p |
| --- | --- | --- | --- | --- |
| a | 0.0003 | 4.12E-05 | 6.77 | <0.0001 |
| h | 0.3100 | 0.0137 | 22.69 | <0.0001 |
| I_max_ (1/h) | 1682 |  |  |  |

AICc = -53.49

## Table S6C. Results of the functional response type II model fit using log-initial prey abundances and grazing coefficients (g) that were not corrected for changes in prey cell numbers in controls. The Hill exponent (ϴ) was 1.2 (see Table 1 and main text for details).

| Parameter | Value | SE | t | p |
| --- | --- | --- | --- | --- |
| a | 0.0001 | 1.92E-05 | 6.381 | <0.0001 |
| h | 0.3181 | 0.0135 | 23.487 | <0.0001 |
| I_max_ (1/h) | 1392 |  |  |  |

AICc = -52.17
